# Normal Predicted Reference Values for Spirometry in Korean Children and Adolescents

**DOI:** 10.3390/children7090105

**Published:** 2020-08-19

**Authors:** Dong Hyun Kim, Jeong Hee Kim, Dae Hyun Lim

**Affiliations:** Department of Pediatrics, Inha University School of Medicine, Inha University Hospital, Incheon 22332, Korea; id@inha.ac.kr (D.H.K.); kimjhmd@inha.ac.kr (J.H.K.)

**Keywords:** pulmonary function test, forced vital capacity, reference value, children, adolescents

## Abstract

Pulmonary function tests are useful to evaluate airway obstructions and bronchial responsiveness. We aimed to determine the reference values applicable to Korean children and adolescents. In total, 5590 (2607 males, 2983 females) healthy children aged 4 to 17 years old were recruited from three regions in Korea. Simple and multiple regression analyses were applied using age, height, and weight as variables to predict the forced vital capacity (FVC), forced expiratory volume in one second (FEV_1_), maximum mid-expiratory flow (MMEF) and the peak expiratory flow rate (PEFR). There were significant correlations between the variables and parameters (*P* < 0.001). The coefficient of determination (R^2^) values of polynomial equations with two variables were lower than those with two variables but higher than those of monomial equations based on height. The prediction equations by height were obtained, and the R^2^ value of the FEV_1_ was the highest. The predicted spirometric values for males were higher than those for females except for the MMEF. The R^2^ values for the FEV1 and FVC were higher than previous studies except for the R^2^ value of the FVC for males in European data. This study provided updated regression equations of normal predicted values for spirometry applicable to Korean children and adolescents.

## 1. Introduction

Pulmonary function tests are very useful to evaluate airway obstructions and bronchial responsiveness. Pulmonary function testing for childhood has increased as respiratory diseases have increased in children. The usefulness of spirometers for assessing respiratory diseases in childhood has emerged because the American Thoracic Society and European Respiratory Society (ATS/ERS) [1] have presented methods and techniques of spirometry and more convenient instruments have been developed.

It is necessary to obtain appropriate reference values for the assessment of results expressed as a fraction of the reference values. The reference values have differences in races, regions and socioeconomic environment. Age, sex and anthropometric factors, such as height and weight, affect reference values especially in childhood [2]. There have been several reports to obtain reference equations for Korean children since the early 1990s [3,4,5,6,7]. However, most of their subjects were recruited in the same region and the number of subjects was small. Their results didn’t seem to be related, so they could not represent all Korean children. Besides, there have been changes in the anthropometric data in Korean children over recent years. The standard data of children’s growth are revised every ten years in Korea. Updated reference values reflecting recent anthropometric data are needed.

This study has been conducted to provide calculable regression equations for spirometric reference values in Korean healthy children and adolescents recruited for four years. Moreover, the study shows an evaluation of the differences from previous Korean data and comparisons with Asian and Caucasian data.

## 2. Materials and Methods

Spirometry was performed in 22,778 survey participants aged 4 to 17 years old from 2010 to 2014. Children were recruited from kindergartens, elementary, middle, and high schools in 4 Korean regions (metropolitan—Gyeonggi, Incheon; southern coast city—Busan; southern inland—Gwangju). They answered questionnaires including a range of questions about respiratory symptoms, and their history of respiratory disease or allergic disease. Some were excluded because the following applied: missing values of information on age, anthropometric data and spirometric data, history of asthma, current cold, age under 4, above 17, upper 3% and lower 3% of the Korean-children physical developmental standard from 2007, and outliers in the spirometry and statistical process. The total number of children was 5590 (2607 males, 2983 females). Their height and weight were measured by one technician and their body surface area (BSA) was converted using the following formula [8].

$$\sqrt{Weight\left(kg\right)\times Height\left(cm\right)÷3600}$$

Pulmonary function tests were performed using a Microplus Spirometer (Carefusion, Kent, UK). Subjects performed according to ATS/ERS (American Thoracic Society/European Respiratory Society) criteria [1] by one experienced technician. The spirometric values of the forced vital capacity (FVC), forced expiratory volume in one second (FEV_1_), maximum mid-expiratory flow (MMEF) and peak expiratory flow rate (PEFR) were recorded.

Simple and multiple regression models were applied between the spirometric data and variables. Variance inflation factors were used for selecting variables. Cook’s distance and r student were used for evaluating outliers. Polynomial equations and the interaction between height and age were also considered. The appropriate regression model was chosen based on the highest explained variation of the dependent variable, adjusted determinant coefficients (R^2^) and simplicity. The regression model includes the natural logarithmic values of spirometric values. All the analyses were performed using SAS 9.4 (SAS Institute Inc., Cary, NC, USA).

Written informed consent was obtained from the parents or guardians of each child. This study was approved by the institutional review board (IRB) of Inha University Hospital (IRB Number, IUH-IRB-12-1222).

## 3. Results

### 3.1. Demographic Characteristics

**Figure 1:**
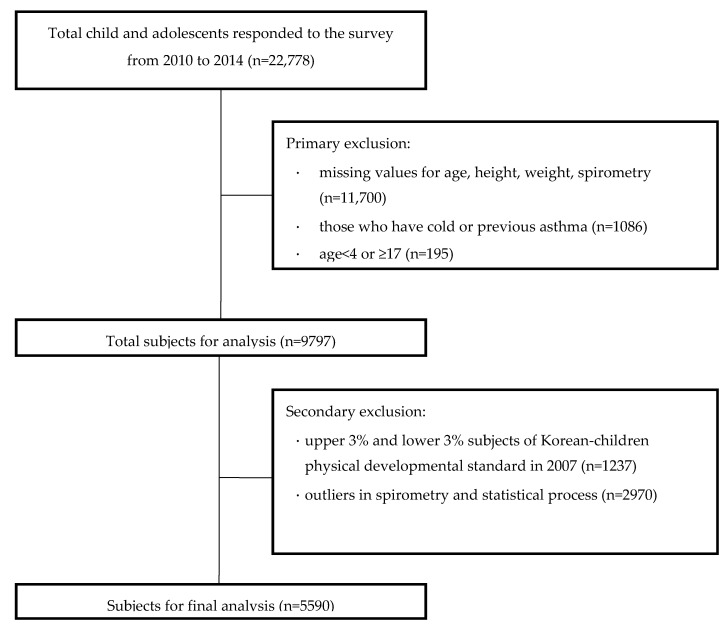
Study profile.

**Figure 2:**
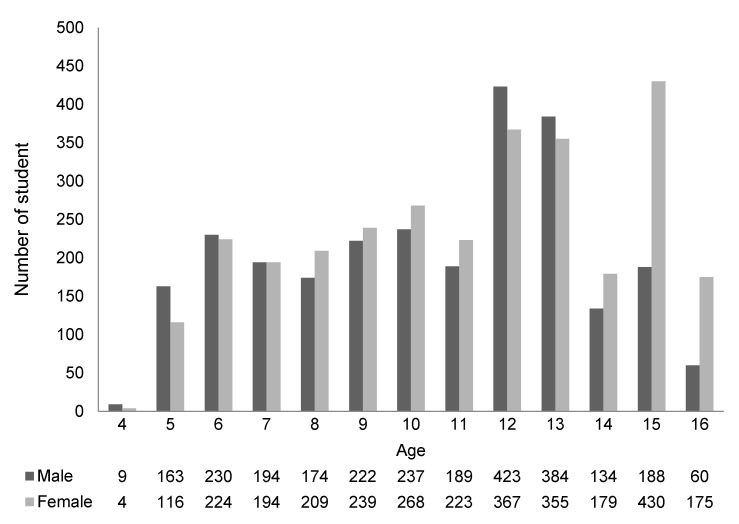
Age distribution of subjects by sex.

In total, 5590 participants were tested and Figure 1 shows the study profile. The analysis was based on 2607 males and 2983 females. The age range of the subjects was 4 to 16 years in both males and females. Figure 2 shows the age distribution of subjects by sex. The most represented age group in males was 12 years old, but 15 years old in females. The least represented age group in both males and females was children who were 4 years old. Table 1 indicates the physical characteristics of subjects by sex. The average height was 144.2 ± 18.1 cm in males and 144.4 ± 16.0 cm in females. The average weight was 40.9 ± 14.5 kg in males and 40.7 ± 12.9 kg in females. There were no significant differences in the height and weight between males and females (Table S1).

### 3.2. Analysis of Spirometric Values

A simple regression analysis of variables and spirometric values was conducted (Table 2). The value of R^2^ for height was highest.

The scatter graphs of spirometric values by height were made and linear regression equations were obtained with a natural logarithmic transformation (Figures S1–S4). The coefficient of determination value of FEV_1_ was the highest. The FVC, MMEF and PEFR were listed in descending order. The R^2^ values of spirometric values for males were higher than those for females. The predicted spirometric values for males were higher than those for females except for MMEF. The predicted value of MMEF for females was the same or higher than that for males.

A multiple regression analysis of spirometric values with three variables was conducted (Table 3). For males, the R^2^ values of FEV_1_ and FVC were higher than those of simple regression analysis on height. However, the R^2^ values of the MMEF and PEFR were lower. For females, the R^2^ values of the FEV_1_, FVC, PEFR were higher than those of simple regression analysis on height, but the R^2^ values of MMEF were lower. Height and weight were selected using variance inflation factors, and polynomial equations with two variables were obtained. The R^2^ values of polynomial equations with height and weight were lower than those with height, BSA, and age but higher than those of monomial equation based on height. However, the R^2^ values of those equations wouldn’t make a difference.

### 3.3. Comparison with Previous Research

This study presented a comparison with previous Korean studies and foreign studies (Figures S5 and S6). Table 4 shows the comparison of predicted values of the FEV1 and FVC with previous five Korean studies. The predicted values at 120 cm and 160 cm were also compared. Table 5 and Figure S8 show the comparison of predicted values of the FEV1 and FVC with six foreign studies. There were three Caucasian studies [9,10,11] and three Asian studies [12,13,14]. Two Caucasian studies [9,10] and one Asian study [14] provided regression equations with two variables—height and age. One Caucasian study [11] and two Asian studies [12,13] provided a regression equation with one variable—height. The polynomial and monomial equations were compared separately.

## 4. Discussions

Spirometric values are affected by individual factors such as height, age, sex, activity level, ethnicity and amount of muscles, environmental factors such as smoking, occupation, residence and air pollution, and technical factors such as spirometers, subjects, technicians and techniques [2,15,16]. In many countries, various studies evaluated the relationships between variables and spirometric values, and most of them said height was most related to spirometric values [3,4,5,6,7,9,10,11,12,13,14].

This study provided updated regression equations of spirometric values with height for healthy Korean children. The subjects recruited were healthy Korean children from four different regions in Korea from 2010 to 2014. The height was the most important factor to predict the spirometric values, similarly to previous studies. Scatter graphs of the spirometric values with height were drawn, but their shapes were not linear. A transformation was considered to make the graph linear. A natural logarithmic transformation was applied to the equations, so that the values of R^2^ were enhanced. A multiple regression analysis was conducted but the values of R^2^ did not differ from equations with a single variable and equations with multivariables were complicated. Therefore, monomial regression equations were selected. In this study, R^2^ was considered important for a comparison with previous studies, assuming that it could contribute to predicting the pulmonary function parameters in a healthy population.

Compared with the spirometric data of Korean children, the values of R^2^ were highest and the predicted values were different to each other (Table 4). These differences are likely due to the difference of study profiling and processing techniques of regression equations. The largest number of subjects was recruited and subjects were recruited from several regions in this study. This study included preschool students aged four and five unlike the others. The focus was on standard healthy children and the outliers were excluded by the residual analysis. The logarithmic transformation enhanced the value of R^2^. Different regions could make differences in the predicted values. The small number of subjects and different age distributions could be the reason for the differences in the same region. Quanjer et al. reported that differences in the spirometric values between centers seemed to be mainly due to sampling errors [17]. They recommended collecting data on at least 300 healthy subjects to validate the reference equations for spirometry. Otherwise, technical factors could affect the results. ATS/ERS criteria1 were applied in this study but not in other studies. Hankinson et al. [9] indicated that differences in the application of standards and instruments could make the values for the FEV_1_ and FVC change.

Compared with foreign studies, there were some differences in regression equations, the value of R2 and the predicted values of the FEV_1_ and FVC (Figure S6). Hankinson et al. [9], Quanjer et al. [10] and Takase et al. [14] provided polynomial equations with height and age, and their values of R^2^ were high. On the other hand, monomial equations were provided in this study. This study also conducted a multiple regression analysis and considered interaction between variables. There were slight differences in the values of R^2^ but the equations are complicated. For both males and females, the values of R^2^ of the FEV_1_ were highest. The R^2^ values for the FVC from Quanjer et al. [10] were highest for males but from Takase et al. [14] the R^2^ values were highest for females. The predicted values of the FEV_1_ and FVC were lower than those from Caucasian data of Hankinson et al. [9] and Quanjer et al. [10] and the Asian data of Takase et al. [14] using polynomial models. The values from Hankinson et al. [9] and Quanjer et al. [10] were similar but from Takase et al. [14] were lower than those from Hankinson et al. [9] and Quanjer et al. [10]. The predicted values of the FEV_1_ and FVC were lower than those from the Caucasian data of Torres et al. [11] and the Asian data of Ip et al. [12] on polynomial models but higher than those from the Asian data of Jeng et al. [13]. The values from Torres et al. [11] and Ip et al. [12] were similar.

Racial differences in lung function have been reported. The lung volume capacity of Korean children was found to be similar to that of Japanese and Chinese but lower than that of American children [7]. The FEV_1_ and FVC in Caucasians were found to be larger than in Chinese and Indians. The larger volume in Caucasians contributed to increased numbers of alveoli and physically larger chest cavities [18]. Africans were found to have a lower volume for FEV_1_ and FVC than that of Caucasians but a higher volume than that of Hispanics [19]. These differences may be due to having a smaller trunk:leg ratio than Caucasians [13]. The lower values observed for Hispanics were attributable to the shorter heights when compared with Caucasian subjects [9]. This study graphed the spirometric values adjusted to height, and the angles under 150 cm were smaller than those over 150cm. The lower values observed for Korean subjects were attributable to the shorter heights when compared with Caucasian subjects. Differences in other Asian studies were attributable to the different age distributions. Asian subjects from Takase et al. [14] and Ip et al. [12] were aged 6 to 18 and 7 to 19, so that the slopes of their equations were steeper than those of this study. Asian subjects from Jeng et al. [13], however, were aged 3–6, so that the slopes were sharper than those of this study. The authors are not sure if there were technical difficulties in the examination of the low coefficient of determination in the studies of Jeng et al. [13]. This study did not exclude the results of the younger age group, which requires a higher proficiency of the technician and has been properly performed.

## 5. Conclusions

This study provided updated regression equations for spirometric values applicable to the Korean children and adolescents. A large sample size with rigorous filtering for samples was likely to reduce sampling errors and reflect a representative population. The equations reflected recent Korean children’s physical growth. It is important to provide new equations for spirometric values as the anthropometric values for children are changing.

## Figures and Tables

**Table 1:** Physical characteristics of subjects by sex.

Sex	N	Height (cm)			Weight (kg)		
		Mean	SD	*p* Value	Mean	SD	*p* Value
Males	2607	144.2	18.1	0.6342	40.9	14.5	0.5432
Females	2983	144.4	16.0		40.7	12.9	
Total	5590	144.3	17.0		40.8	13.7	

N, number; SD, standard deviation.

**Table 2:** Simple regression coefficients for predicting the spirometric parameters.

	FEV_1_		FVC		MMEF		PEFR	
Variables	Constant	β (R^2^)	Constant	β (R^2^)	Constant	β (R^2^)	Constant	β (R^2^)
**Males**								
Ht	−2.0609	0.0191 (0.9274)	−2.0908	0.0199 (0.9113)	−1.4568	0.0163 (0.7153)	−1.0434	0.0170 (0.7983)
Wt	−0.1922	0.0217 (0.7609)	−0.1478	0.0227 (0.7555)	0.1314	0.0185 (0.5895)	0.6240	0.0193 (0.6516)
BSA	−0.7059	1.1017 (0.8483)	−0.6839	1.1527 (0.8399)	−0.3045	0.9377 (0.6548)	0.1657	0.9807 (0.7285)
Age	−0.5031	0.1097 (0.8733)	−0.4642	0.1141 (0.8543)	−0.1414	0.0943 (0.6867)	0.3331	0.0989 (0.7686)
**Females**								
Ht	−2.1195	0.0192 (0.9048)	−2.1040	0.0195 (0.8802)	−1.5906	0.0173 (0.6863)	−1.1603	0.0174 (0.7358)
Wt	−0.2510	0.0222 (0.7843)	−0.2063	0.0226 (0.7666)	0.0957	0.0201 (0.5977)	0.5369	0.0201 (0.6353)
BSA	−0.7484	1.1037 (0.8527)	−0.7125	1.1244 (0.8326)	−0.3527	0.9979 (0.6477)	0.0860	0.9999 (0.6911)
Age	−0.3992	0.0911 (0.8174)	−0.3496	0.0922 (0.7875)	−0.0529	0.0837 (0.6419)	0.3896	0.0836 (0.6804)

*p* < 0.001 for all correlation coefficients, FEV_1_, forced expiratory volume in one second; FVC, forced vital capacity; MMEF, maximal mid-expiratory flow; PEFR, peak expiratory flow rate; β, beta coefficient; R^2^, coefficient of determination; Ht, height (cm); Wt, weight (kg); BSA, body surface area (m^2^).

**Table 3:** Multiple regression coefficients and constants for the predicted spirometric parameters.

	Variables	ln FEV_1_	ln FVC	ln MMEF	ln PEFR
Males	Constant	−10.6765	−10.4462	−8.6598	−7.2801
	ln Ht	2.2362	2.2024	1.8576	1.6281
	ln BSA	0.1126	0.1908	0.0597	0.0992
	ln Age	0.1056	0.1088	0.1340	0.2555
	MSE	0.0093	0.0121	0.0352	0.0232
	Adj. R^2^	0.9283	0.9153	0.7104	0.8058
Females	Constant	−8.4244	−8.3855	−6.8313	−6.4187
	ln Height	1.7500	1.7636	1.4524	1.4526
	ln BSA	0.2964	0.3451	0.2237	0.2186
	ln Age	0.1342	0.1114	0.2015	0.2136
	MSE	0.0096	0.0126	0.0355	0.0275
	Adj. R^2^	0.9081	0.8863	0.6844	0.7406

*p* < 0.001 for all correlation coefficients, FEV_1_, forced expiratory volume in one second; FVC, forced vital capacity; MMEF, maximal mid-expiratory flow; PEFR, peak expiratory flow rate; Adj. R^2^, adjusted coefficient of determination; Ht, height (cm); MSE, mean squared error.

**Table 4:** Comparison of predicted values of the FEV_1_ and FVC with previous Korean data.

	Sex	Author	N	Age	Regression Equation	r^2^ (r)	Value at 120 cm (L)	Value at 160 cm (L)
FEV_1_	Males	Yoon et al. (1993) [3]	1056	6–15	FEV_1_ = −3.277 + 0.037 × Ht	0.8114	1.163	2.643
		Lim et al. (1994) [4]	490	6–11	FEV_1_ = −2.391 + 0.031 × Ht	(0.82)	1.329	2.569
		Nam et al. (1998) [5]	282	8–18	FEV_1_ = −5.057 + 0.049 × Ht	0.796	0.823	2.783
		Song et al. (2002) [6]	716	6–15	FEV_1_ = −3.497 + 0.0396 × Ht	0.861	1.255	2.839
		Park et al. (2014) [7]	189	6–12	FEV_1_ = −2.753 + 0.033 × Ht	0.818	1.207	2.527
		This study	2607	4–16	FEV_1_ = −2.0609 + 0.0191 × Ht	0.9274 * (0.9490)	1.260	2.705
	Females	Yoon et al. (1993) [3]	966	6–15	FEV_1_ = −2.666 + 0.0319 × Ht	0.7628	1.162	2.438
		Lim et al. (1994) [4]	475	6–11	FEV_1_ = −2.505 + 0.031 × Ht	(0.84)	1.215	2.455
		Nam et al. (1998) [5]	170	8–18	FEV_1_ = −2.941 + 0.033 × Ht	0.612	1.019	2.339
		Song et al. (2002) [6]	601	6–15	FEV_1_ = −2.985 + 0.0351 × Ht	0.883	1.227	2.631
		Park et al. (2014) [7]	217	6–12	FEV_1_ = −2.939 + 0.034 × Ht	0.805	1.141	2.501
		This study	2983	4–16	lnFEV_1_ = −2.1195 + 0.0192 × Ht	0.9048 * (0.9299)	1.203	2.592
FVC	Males	Yoon et al. (1993) [3]	1056	6–15	FVC = −4.088 + 0.046 × Ht	0.7547	1.432	3.272
		Lim et al. (1994) [4]	490	6–11	FVC = −2.636 + 0.034 × Ht	(0.78)	1.444	2.804
		Nam et al. (1998) [5]	282	8–18	FVC = −5.472 + 0.053 × Ht	0.814	0.888	3.008
		Song et al. (2002) [6]	716	6–15	FVC = −3.556 + 0.0407 × Ht	0.848	1.328	2.956
		Park et al. (2014) [7]	189	6–12	FVC = −3.018 + 0.036 × Ht	0.791	1.302	2.742
		This study	2607	4–16	ln FVC = −2.0908 + 0.0199 × Ht	0.9113 * (0.9399)	1.346	2.984
	Females	Yoon et al. (1993) [3]	966	6–15	FVC = −3.696 + 0.0423 × Ht	0.698	1.38	3.072
		Lim et al. (1994) [4]	475	6–11	FVC = −2.878 + 0.035 × Ht	(0.83)	1.322	2.722
		Nam et al. (1998) [5]	170	8–18	FVC = −3.533 + 0.038 × Ht	0.637	1.027	2.547
		Song et al. (2002) [6]	601	6–15	FVC = −3.054 + 0.036 × Ht	0.872	1.266	2.706
		Park et al. (2014) [7]	217	6–12	FVC = −3.087 + 0.036 × Ht	0.799	1.233	2.673
		This study	2983	4–16	in FVC = −2.1040 + 0.0195 × Ht	0.8802 * (0.9131)	1.266	2.762

N, number; H, height (cm); r, correlation coefficient; r^2^, coefficient of determination; *, adjusted coefficient of determination. FEV_1_, forced expiratory volume in one second; FVC, forced vital capacity; MMEF, maximal mid-expiratory flow; PEFR, peak expiratory flow rate.

**Table 5:** Comparison of predicted values of the FEV_1_ and FVC with previous foreign data.

	Sex	Author	N	Age	Regression Equation	r^2^
FEV_1_	Males	Hankinson et al. (1999) [9]	422	8–20	FEV_1_ = −0.7453 − 0.04106 × A + 0.004477 × A^2^ + 0.00014098 × Ht^2^	0.85
		Quanjer et al. (1995) [10]	3592	6–21	ln FEV_1_ = −1.2933 + (1.2669 + 0.0174A) × Ht/100	0.92
		Ip et al. (2000) [12]	392	7–19	ln FEV_1_ = −13.999 + 2.972 × ln Ht	0.9
		Torres et al. (2003) [11]	48	6–10	FEV_1_ = −2.1413 + 0.0285 × Ht	0.6672
		Jeng et al. (2009) [13]	109	3–6	FEV_1_ = −1.372 + 0.0221 × Ht	0.448
		Takase et al. (2013) [14]	674	6–18	FEV_1_ = 3.347 −0.1174 × A + 0.0079 × A^2^ − 4.831 × Ht/100 + 2.997 × (Ht/100)^2^	0.919
		This study	2607	4–16	ln FEV_1_ = −2.0609 + 0.0191 × Ht	0.9274 *
	Females	Hankinson et al. (1999) [9]		8–18	FEV_1_ = −0.8710 + 0.06537 × A + 0.00011496 × Ht^2^	0.75
		Quanjer et al. (1995) [10]	2269	6–21	ln FEV_1_ = −1.5974 + (1.5016 + 0.0119 × A) × H/100	0.88
		Ip et al. (2000) [12]	460	7–19	ln FEV_1_ = −13.392 + 2.843 × ln H	0.79
		Torres et al. (2003) [11]	52	6–10	FEV_1_ = −2.1413 + 0.0285 × Ht	0.6672
		Jeng et al. (2009) [13]	105	3–6	FEV_1_ = −1.381 + 0.0219 × Ht	0.578
		Takase et al. (2013) [14]	622	6–18	FEV_1_ = 1.842 + 0.00161 × A^2^ − 3.354 × Ht/100 + 2.357 × (Ht/100)^2^	0.857
		This study	2983	4–16	ln FEV_1_ = −2.1195 + 0.0192 × Ht	0.9048 *
FVC	Males	Hankinson et al. (1999) [9]	422	8–20	FEV_1_ = −0.2584 − 0.20415 × A + 0.010133 × A^2^ + 0.00018642 × H^2^	0.85
		Quanjer et al. (1995) [10]	3592	6–21	ln FEV_1_ = −1.2782 + (1.3731 + 0.0164 × A) × Ht/100	0.93
		Ip et al. (2000) [12]	392	7–19	ln FEV_1_ = −13.851 + 2.964 × ln Ht	0.9
		Torres et al. (2003) [11]	48	6–10	FVC = −3.2005 + 0.039 × H	0.7203
		Jeng et al. (2009) [13]	109	3–6	FVC = −1.629 + 0.0251 × H	0.477
		Takase et al. (2013) [14]	674	6–18	FVC = 2.108 − 0.1262 × A + 0.00819 × A^2^ − 3.118 × Ht/100 + 2.553 × (Ht/100)^2^	0.912
		This study	2607	4–16	ln FVC = −2.0908 + 0.0199 × H	0.9113 *
	Females	Hankinson et al. (1999) [9]		8–18	FVC = −1.2082 + 0.05916 × A + 0.00014815 × Ht^2^	0.73
		Quanjer et al. (1995) [10]	2269	6–21	ln FVC = −1.4507 + (1.4800 + 0.0127 × A) × H/100	0.88
		Ip et al. (2000) [12]	460	7–19	ln FVC = −13.270 + 2.835 × ln H	0.79
		Torres et al. (2003) [11]	52	6–10	FVC = −2.4448 + 0.0322 × H	0.6867
		Jeng et al. (2009) [13]	105	3–6	FVC = −1.661 + 0.0250 × H	0.614
		Takase et al. (2013) [14]	622	6–18	FVC = 1.142 + 0.00168 × A^2^ − 2.374 × Ht/100 + 2.116 × (Ht/100)^2^	0.842
		This study	2983	4–16	ln FVC = −2.1040 + 0.0195 × H	0.8802 *

N, number; Ht, height (cm); A, age; r^2^, coefficient of determination; *, adjusted coefficient of determination. FEV_1_, forced expiratory volume in one second; FVC, forced vital capacity; MMEF, maximal mid-expiratory flow; PEFR, peak expiratory flow rate.

## Supplementary Materials

The following are available online at https://www.mdpi.com/2227-9067/7/9/105/s1, Figure S1: Relationship of ln FEV_1_ to height, Figure S2: Relationship of ln FVC to height, Figure S3: Relationship of ln MMEF to height, Figure S4: Relationship of ln PEFR to height, Figure S5: Comparison of the FEV_1_ according to height in males and females, Figure S6: Comparison of the FVC according to height in males and females, Figure S7: Comparison of the FEV_1_ according to height in males and females, Figure S8: Comparison of the FVC according to height in males and females, Table S1: Physical characteristics of subjects by regions.
