# Supplementary material for: Normal Predicted Reference Values for Spirometry in Korean Children and Adolescents
Source: Children (Basel). 2020 Aug 19;7(9):105. doi: 10.3390/children7090105 (PMC7552629; doi:10.3390/children7090105)
Supplement: Supplementary file 1 [file children-07-00105-s001.pdf]

SUPPLEMENTS

Table S1 Physical Characteristics of Subjects by Regions

Region	N	Height(cm)			Weight(kg)		
		Mean	STD	P value	Mean	STD	P value
Metropolitan	3,435	144.9	17.3	<0.0001	41.6	13.9	<0.0001
Southern coast city	1,237	141.9	17.1		38.5	13.3	
Southern inland	918	145.3	15.6		40.8	12.7	
Total	5,590	144.3	17.0		40.8	13.7	

Metropolitan: Gyeonggi, Incheon; Southern coast city: Busan; Southern inland: Gwangju; N, number; STD, standard deviation

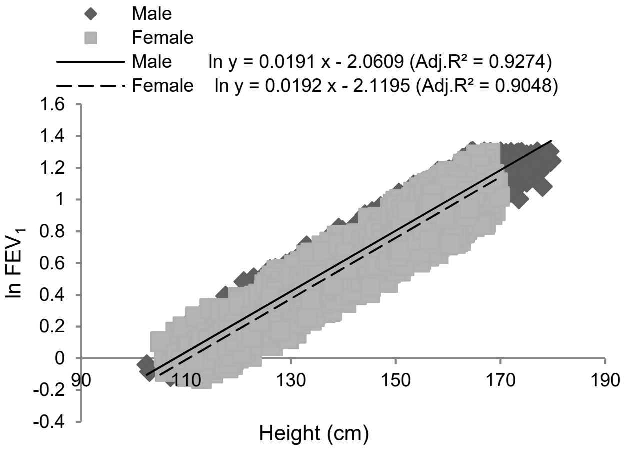

Figure S1 Relationship of ln FEV1 to Height

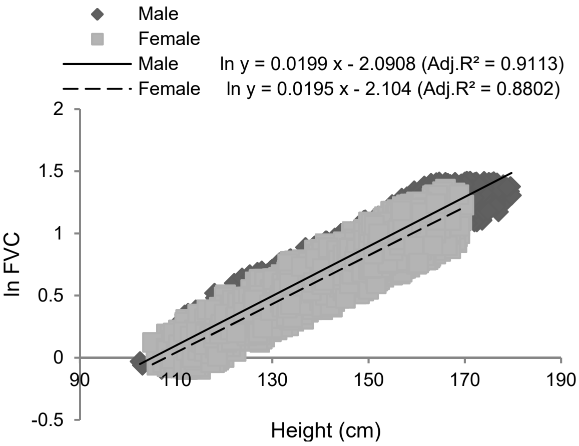

Figure S2 Relationship of ln FVC to Height

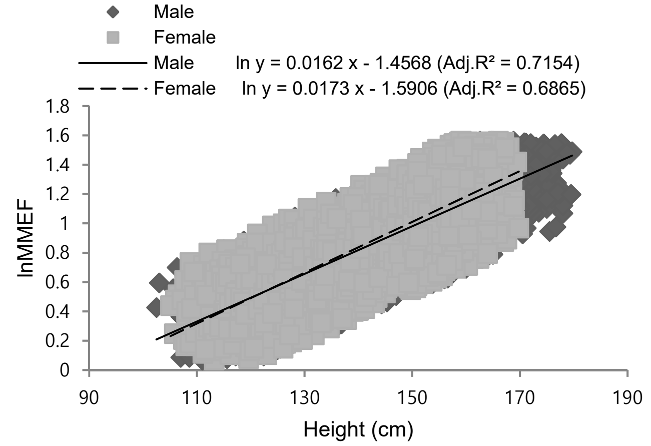

Figure S3 Relationship of ln MMEF to Height

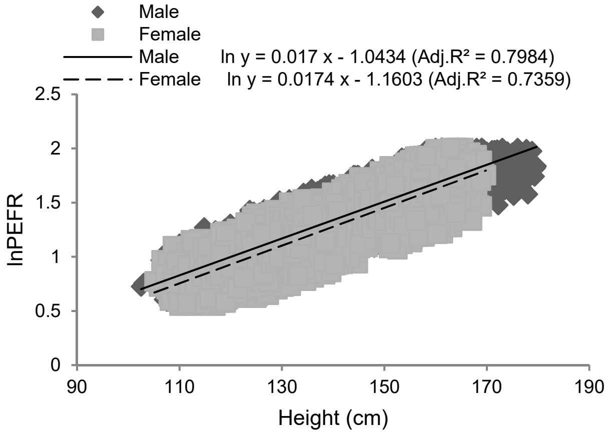

Figure S4 Relationship of ln PEFR to Height

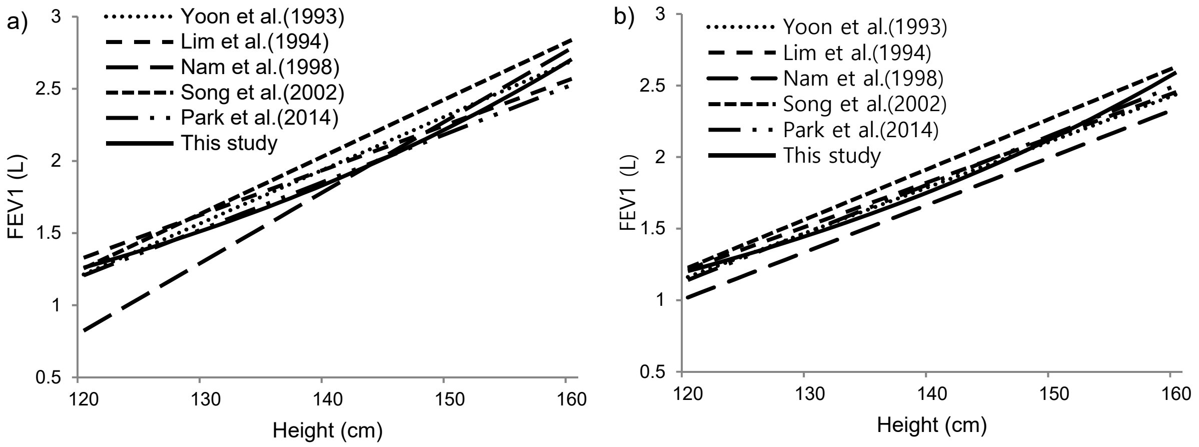

Figure S5 Comparison of FEV_1 according to Height in Male(a) and Female(b)

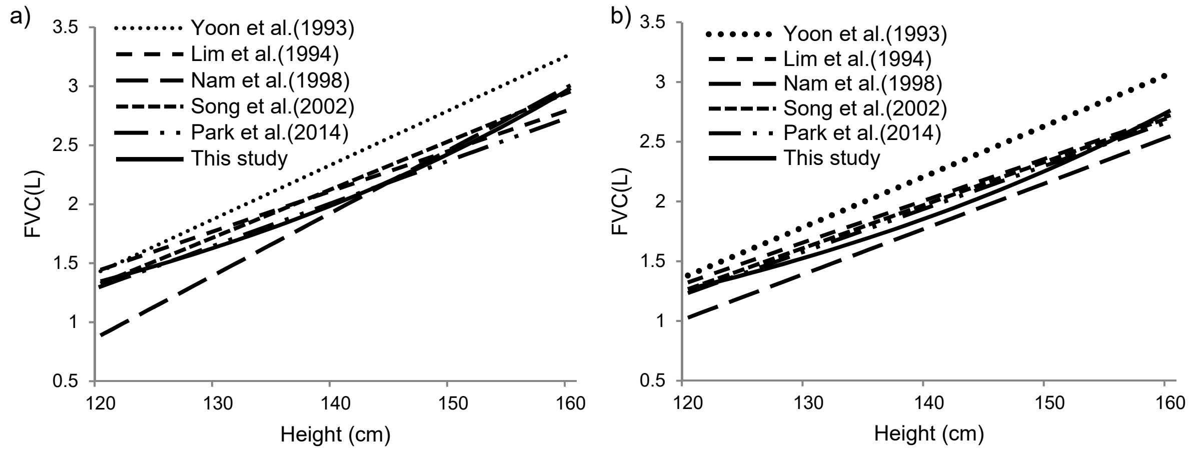

Figure S6 Comparison of FVC according to Height in Male(a) and Female(b)

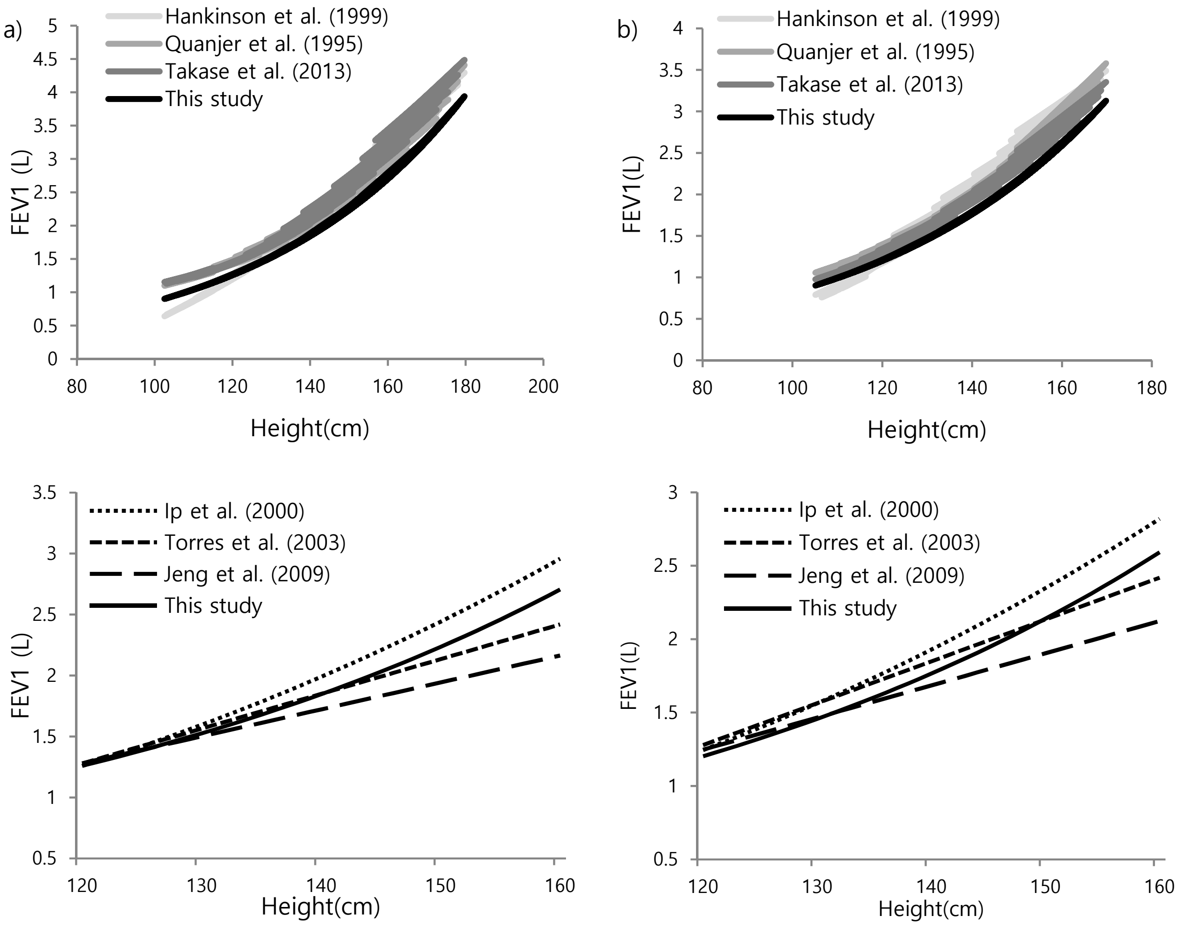

Figure S7 Comparison of FEV_1 according to height in male(a) and female(b)

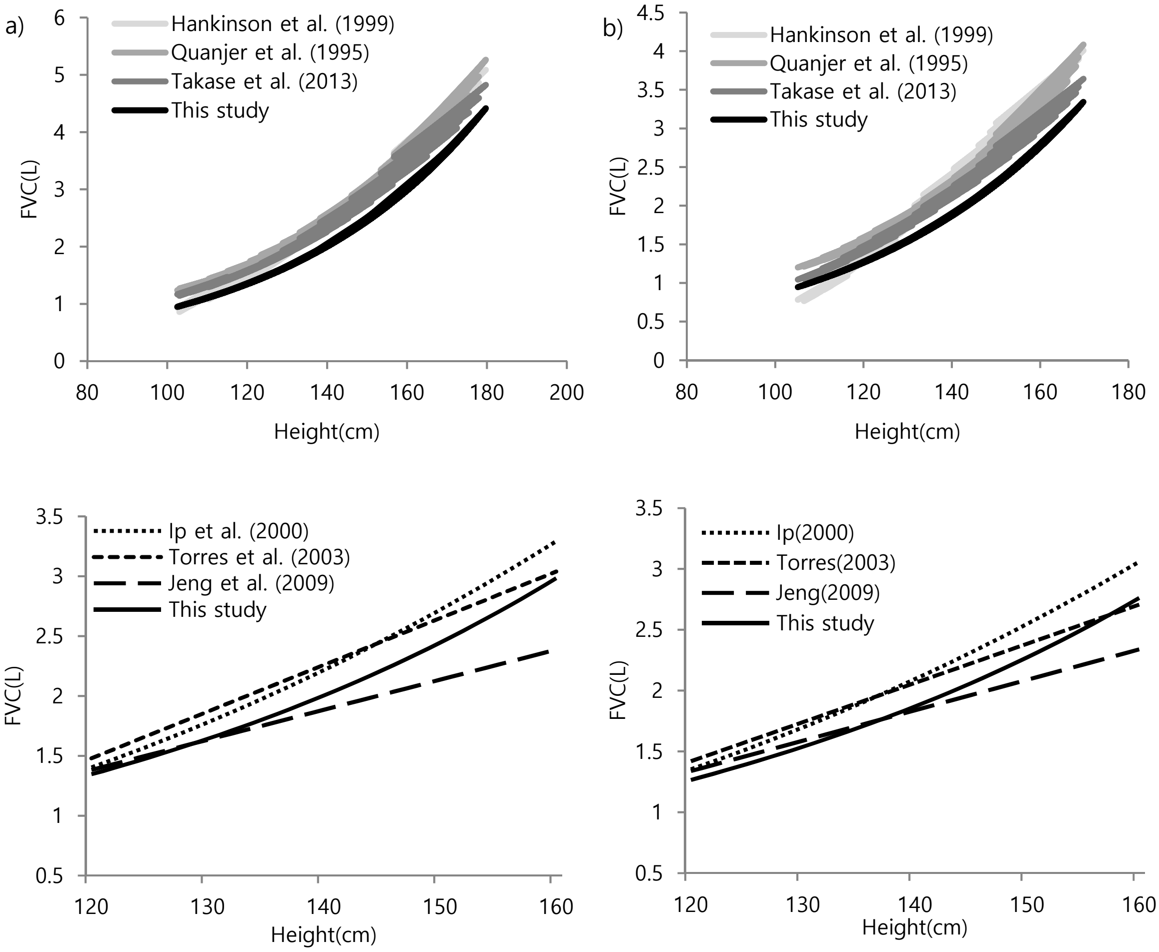

Figure S8 Comparison of FVC according to Height in Male(a) and Female(b)
